# Evaluation of transmission characteristics of CVD-grown graphene and effect of tuning electrical properties of graphene up to 50 GHz

**DOI:** 10.1038/s41598-023-40942-8

**Published:** 2023-08-24

**Authors:** Ryota Okuda, Kazuhiko Niwano, Kaname Hatada, Kei Kokubu, Ryosuke Suga, Takeshi Watanabe, Shinji Koh

**Affiliations:** 1 Technology General Division, Materials Integration Laboratories, AGC Inc., Yokohama, 230-0045 Japan; 2 Department of Electrical Engineering and Electronics, College of Science and Engineering, Aoyama Gakuin University, Sagamihara, 252-5258 Japan

**Keywords:** Electronic properties and devices, Electrical and electronic engineering

## Abstract

Graphene has been investigated as a transparent conductive film for use in a variety of devices, and in recent years it has shown promise for use in millimeter-wave devices as 5G technology. In this study, we applied single-layer (SL), triple-layer (3L), and P-type doped 3L graphene to coplanar waveguide (CPW) transmission lines and obtained transmission characteristics (S_21_) from 1 to 50 GHz, which covered the 5G band. Furthermore, an equivalent circuit model of the CPW used in the measurements was constructed and simulations were performed, which showed good agreement with the measured results. The results validated the transmission properties of the graphene and the contact impedance at the interface between electrodes and the graphene in CPW circuits, which are necessary parameters for designing antennas using graphene. In addition, by comparing the transmission loss of three types of graphene, the parameters for improving the transmission characteristics were clarified.

## Introduction

In recent years, various conductive materials have been considered for applications of millimeter-wave device as fifth generation (5G) technology^1–3^. In particular, transparent conductive materials are attracting attention because they can be installed in a variety of locations without compromising their appearance. For example, indium tin oxide^4^, indium zinc tin oxide^5^, and meshed Cu formed on a substrate^6^ are commonly used, however, these materials have drawbacks such as trade-offs between electrical resistance and optical transparency^7,8^. Since our previous report, we have been focusing on chemical vapor deposition (CVD) grown graphene^9,10^. In general, graphene has excellent optical transparency and can be transferred onto various shapes and types of substrates due to its robustness and flexibility. If graphene can be implemented as a transparent 5G antenna, it can be embedded in transparent objects that require high optical transparency, such as building windows, car windshields, and mobile phones. However, graphene has higher carrier mobility but lower carrier concentration than metals, resulting in higher electrical resistance. This poses a challenge for its application in millimeter-wave devices. Recently, graphene has been extensively studied as an alternative material to metals, and various studies have been conducted to achieve low resistance while maintaining transparency using methods such as multilayering^11,12^ and carrier doping^13,14^. There have been reports investigating the electrical properties of graphene itself and its application to millimeter-wave devices. Pan et al. fabricated the slot antennas using ink containing graphene and evaluated their radiation characteristics up to 18 GHz^15^. Sa'don et al*.* reported printed array antennas with graphene and their radiation characteristics and beamforming capability at 15 GHz^16^. Hong et al*.* compared the electromagnetic interference (EMI) shielding performance of graphene with different qualities by changing the CVD process parameters^17^. On the other hand, there are limited reports evaluating the characteristics of devices using various types of graphene with different electrical properties in the frequency bands used for 5G technology (24.5 to 27.5 GHz and 37.0 to 43.5 GHz). Zhang et al. have evaluated transmission properties up to 40 GHz by applying graphene to a coplanar waveguide (CPW) with different structures with single layer graphene^18^. Similarly, Moon et al. evaluated the transmission properties of graphene from 0.5–110 GHz with different number of layers^19^. Grande et al. evaluated microwave ring resonators with multilayering and SOCl_2_ doping up to 10 GHz^20^. While device applications of graphene in the terahertz band^21–23^ have attracted much attention, there is insufficient research targeting the millimeter-wave region for 5G applications, which we aim to cover in our study.

In this report, we systematically evaluate the effects of graphene multilayering and carrier doping on transmission characteristics (S_21_) from 1 to 50 GHz. Three types of graphene were fabricated: single-layer (SL), three-layer (3L), and P-type carrier doped 3L graphene with TFSA (Bis (trifluoromethanesulfonyl) amide) as the dopant, and their S_21_ were measured. Furthermore, the electrical properties of graphene in the CPW were clarified by simulating the S_21_ using the constructed equivalent circuit model of the CPW and comparing it with the measured S_21_ of the CPW. The results clarified the behavior of three types of graphene in millimeter-wave devices such as its transmission characteristics and contact characteristics at the interfaces between graphene and electrodes, which are necessary parameter for designing antennas using graphene.

## Results and discussions

In our study, SL, 3L, and P-type doped 3L graphene were fabricated to clarify how the transmission properties of graphene change with the number of layers and carrier doping. The fabrication of graphene and the devices for evaluation are described in the “Methods” section.

### Optical transmittance in visible region

**Figure 1:**
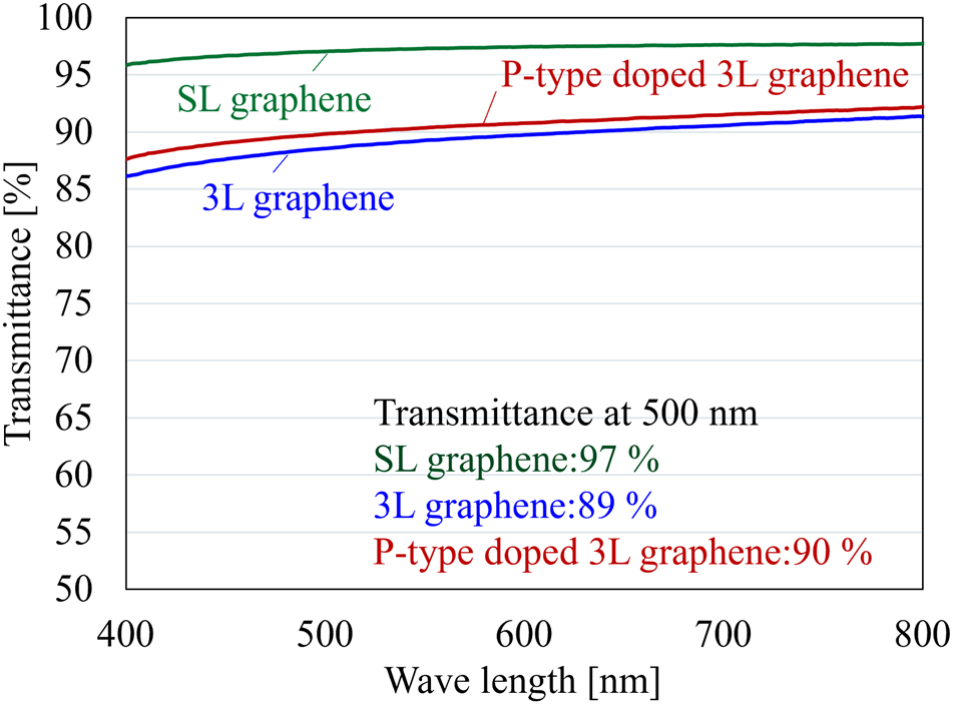
Visible spectral evaluation of SL graphene (green), 3L graphene (blue) and P-type doped 3L graphene (red).

The optical transmittances in the visible region of the SL, 3L, and P-type doped graphene are shown in Fig. 1. Because a certain area (2 × 7 mm^2^) is required to evaluate optical transmittance evaluation, three types of graphene (10 × 10 mm^2^) were transferred to a quartz substrate and evaluated optical properties before forming the device. Furthermore, carrier doping was performed on 3L graphene before device fabrication, and the optical transmittance of P-type doped 3L graphene was evaluated. Note that the optical transmittance of the quartz substrate was subtracted for reference to discuss the optical properties of graphene only. As shown in Fig. 1, SL graphene showed the highest transmittance (97% at 500 nm). Graphene has an absorption of about 2.3% per layer in a visible wavelength region. Therefore, the transmittance of 3L graphene (89% at 500 nm) was lower than that of the SL graphene. Notably, P-type doped 3L graphene exhibited an optical transparency similar to pristine 3L graphene (90% at 500 nm). The P-type doping has the effect of increasing carrier density because it adds more holes, which is expected to improve conductivity^24^. Being able to improve electrical conductivity while maintaining optical transparency is a unique feature of graphene.

### Raman spectra of graphene

**Figure 2:**
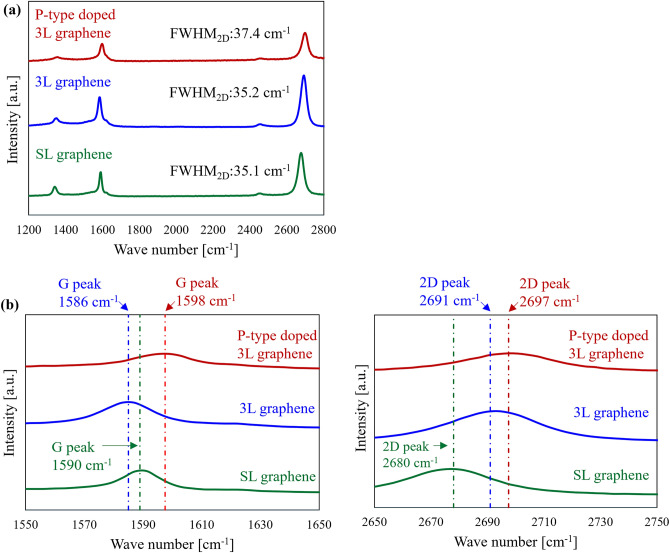
(**a**) Raman spectra of SL graphene (green), 3L graphene (blue) and P-type doped 3L graphene (red) (**b**) Raman shift of SL graphene, 3L graphene and P-type doped 3L graphene of G and 2D peaks.

The Raman spectra of the three types of graphene are shown in Fig. 2. The measurements were performed at the center of the graphene channels of the CPW devices. After evaluating the Raman spectra of 3L graphene, carrier doping was performed on the same sample to obtain the Raman spectra of P-type doped 3L graphene. G peaks at 1580–1600 cm^-1^ and 2D peaks at 2680–2700 cm^-1^, which are characteristic of graphene, were observed as shown in Fig. 2a. For all samples, slight D peaks were observed at 1360–1370 cm^-1^, which may be due to the defects induced during the transferring, stacking and fabricating the CPW processes. Significant damage was not observed with the optical microscope observation in Fig. S1. The intensity ratio of the 2D peak to the G peak in the Raman spectra (I_2D_/I_G_) correlate with the stacking structure of a multilayer graphene. The obtained I_2D_/I_G_ of the 3L graphene was 1.5, which is as high as that of SL graphene (I_2D_/I_G_ = 1.5). As shown in Fig. 2a, the full width at half maximum (FWHM_2D_) from fitting the 2D peak with the Lawrence function indicates that 3L graphene (FWHM_2D_:35.2) is close to SL graphene (FWHM_2D_:35.1). These suggested 3L graphene had a turbostratic stacking structure, which is characteristic of multilayer graphene obtained from a layer-by-layer process using polycrystalline graphene grown on polycrystalline Cu foils^10,18,25^. In general, the G peak of graphene originates from the in-plane motion of carbon atoms and shifts toward higher energies with higher carrier density^26,27^. In Fig. 2b, the G peak of 3L graphene was observed to shift from 1583 to 1598 cm^-1^, which supported P-type doping with TFSA.

### Characterization of transmission characteristics (S_21_) using CPW devices

**Figure 3:**
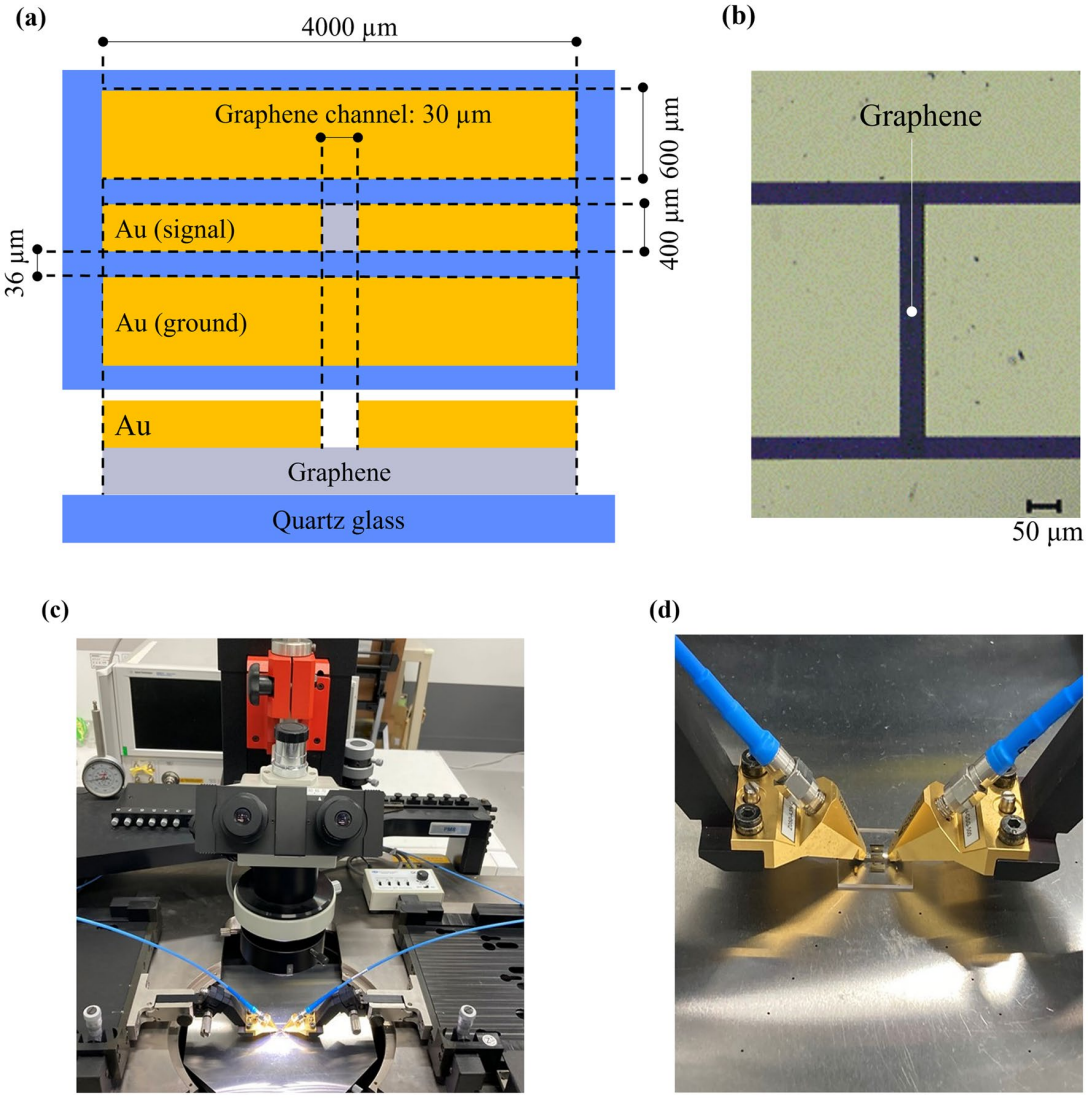
(**a**) Design and (**b**) optical microscope image of CPW. (**c**) Measurement system. (**d**) Probe measurement area.

CPWs were used to evaluate the S_21_ of graphene (Fig. 3a). A CPW consists of a signal line and ground lines and can be mounted on only one side of the board. Therefore, it is suitable for graphene evaluation because three-terminal probe measurements can be easily performed. CPWs with three types of graphene were fabricated by depositing Au as an electrode on the graphene samples and performing lithography. The characteristic impedance of the signal and ground lines was designed to have a characteristic impedance of 50 Ω^28^, with a signal line width of 400 µm and gaps of 36 μm between the signal line and ground lines. The graphene channel was formed to be 30 × 400 μm^2^ (Fig. 3a,b). Note that graphene is under each of the signal and ground lines with Au. The S_21_ of the fabricated CPW was measured from 1 to 50 GHz using a microwave probe (Form Factor, |Z| Probe GSG 500 μm) with guaranteed measurement range from DC to 50 GHz, a probe station (Form Factor, PM8), and a vector network analyzer (Keysight Technologies, E8361A) as shown in Fig. 3c,d. For the S_21_ measurement, the CPW was connected to two ports of the vector network analyzer and input impedance of 50 Ω was extracted. After calibrating shorts, opens, loads, and throughs, the S_21_ was measured.

### Equivalent circuit model of CPW

**Figure 4:**
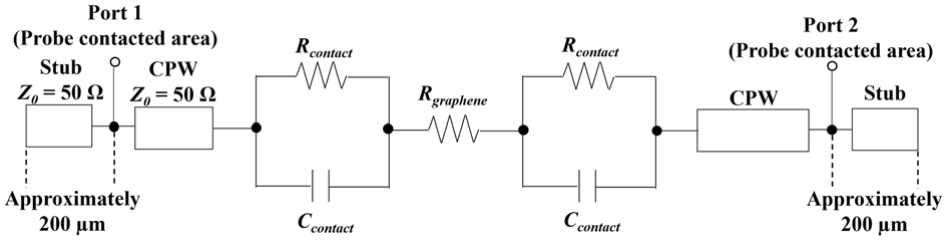
Equivalent circuit model of CPW.

To analyze the contact impedance and the transmission characteristics of the graphene samples, an equivalent circuit model of the CPW was constructed as shown in Fig. 4^29,30^. The contact impedance between graphene and Au in the CPW transmission line was treated as a parallel circuit of contact resistance *R*_*contact*_ and contact capacitance *C*_*contact*_. To prevent delamination of the Au electrodes in the probe measurement, the probe was contacted about 200 µm inside from both ends of the transmission line. For the frequency from 1 to 50 GHz, the areas between the probe contact position and the edge of the transmission line could act as an open stub, so the distance between the probe contacted area and the end of the transmission line was incorporated into the equivalent circuit as a transmission line with a characteristic impedance of 50 Ω (Fig. S2).

The graphene channel in the transmission line was expressed only in terms of frequency-independent impedance as *R*_*graphene*_. In the Drude model, the conductivity of graphene must take into account the frequency dependence associated with carrier scattering. This scattering follows an exponential relaxation time τ (0.01–2 picoseconds) depending on the mobility, Fermi level, and Fermi velocity^31,32^. The characteristic exponential relaxation time of τ = 2 picoseconds corresponds to ω/2π = 5.0 × 10^11^ (500 GHz), which means that the resistor–capacitor (RC) network can be considered constant at least up to 500 GHz^33,34^. Furthermore, the skin effect of the conductor is also frequency dependent. The thickness of the skin effect from typical CVD graphene conductivity (1.0 × 10^6^ S/m) is 2.3–16 μm^35^ from 1 to 50 GHz as calculated using the following equation,

$$d=\sqrt{\frac{2\rho }{\omega \mu }} \left[m\right]$$

which is much thicker than 3L graphene, and the equivalent circuit model was constructed assuming that the frequency dependence of the impedance of the graphene channel in transmission line is negligible^36^. In parallel with *R*_*graphene*_, the parasitic capacitance generation between Au electrodes across graphene channel can also be accounted for, but it was not included in the equivalent circuit model because the electrode thickness (500 nm) is thin and the distance between electrodes across graphene channels (30 μm) in Fig. 3a is presumed to be greater than the distance between Au and graphene.

To assign the impedance such as *R*_*contact*_ and *R*_*graphene*_ of the equivalent circuit model (Fig. 4), experimental measurements were conducted using three types of graphene.

### Impedance of ***R***_***graphene***_

**Figure 5:**
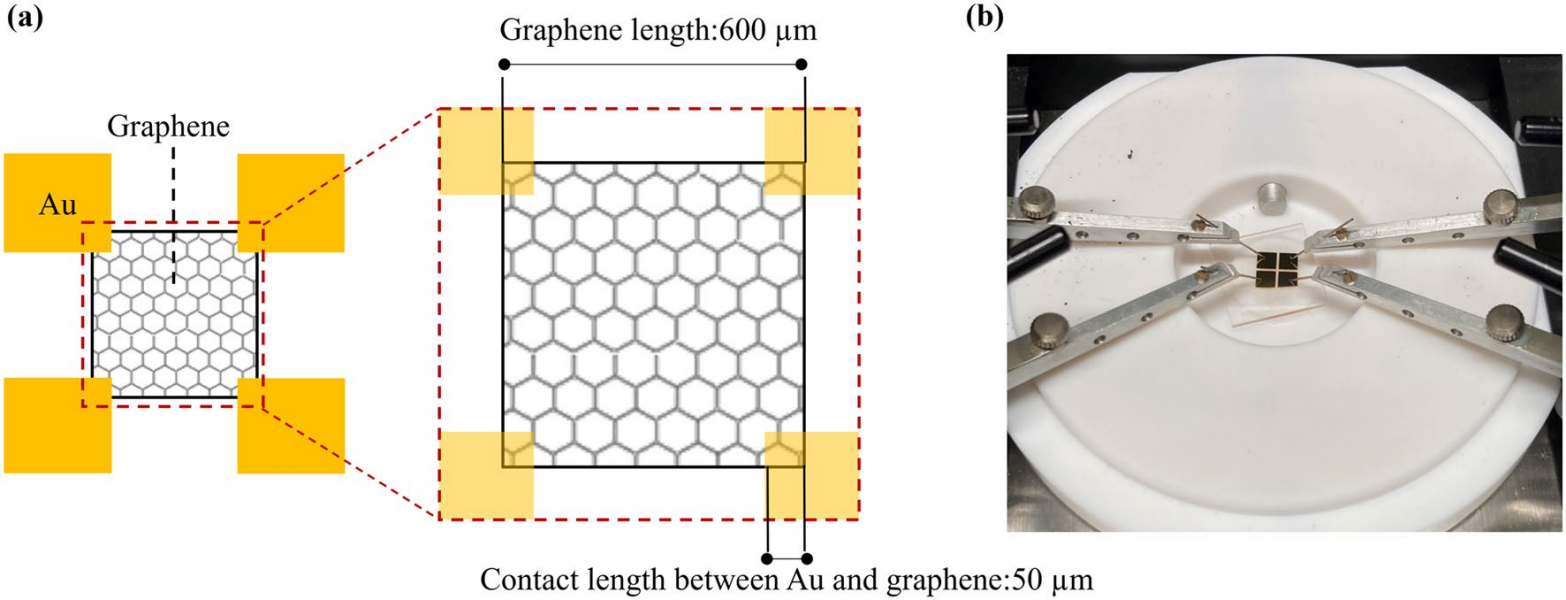
(**a**) Design and (**b**) image of Hall effect measurement.

To determine *R*_*graphene*_, Hall effect measurements were performed on the three types of graphene using the van der Pauw method (Fig. 5a). Such properties can be measured without effects from the contact resistance. The results of the Hall effect measurements (Fig. 5b) are shown in Table 1. The carrier mobility of 3L graphene (2250 cm^2^/Vs) improved compared to that of SL graphene (1200 cm^2^/Vs). This suggests that graphene is less likely to be affected by carrier scattering effects from the quartz substrate due to multilayering^37^. The sheet resistance of graphene (*R*_*sheet*_) was 758 Ω, 405 Ω, and 125 Ω for SL, 3L, and P-type doped 3L graphene, respectively. The carrier mobility (1090 cm^2^/Vs) of P-type doped 3L graphene decreased with TFSA doping, while the sheet resistance decreased with high carrier density (4.6 × 10^13^ cm^-2^). A comparison of the carrier mobility of SL graphene (4.6 × 10^12^ cm^-2^) and 3L graphene (6.8 × 10^12^ cm^-2^) shows that carrier doping is effective in lowering resistivity. *R*_*graphene*_ in the transmission line was determined from *R*_*sheet*_, taking into account the shape of the signal line of the CPW (Fig. 3a) as follows

$$R_{graphene} = R_{sheet} \times \left( \frac{L}{W} \right)$$

where *W* is the length (30 µm) and *L* is the width of the graphene channel in the transmission line (Fig. 3a). As shown in Table 1, the determined *R*_*graphene*_ values were lowest for P-type doped 3L graphene (13 Ω) versus SL (42 Ω) and 3L graphene (78 Ω).

**Table 1:** Results of Hall effect measurement of three types of graphene.

	SL graphene	3L graphene	P-type doped 3L graphene
*R*_*sheet*_ [Ω]	758	405	125
Carrier mobility [cm^2^/Vs]	1200	2250	1090
Carrier density [1/cm^2^]	4.6 × 10^12^	6.8 × 10^12^	4.6 × 10^13^
*R*_*grephene*_ [Ω]	78	42	13

### Contact resistance ***R***_***contact***_

**Figure 6:**
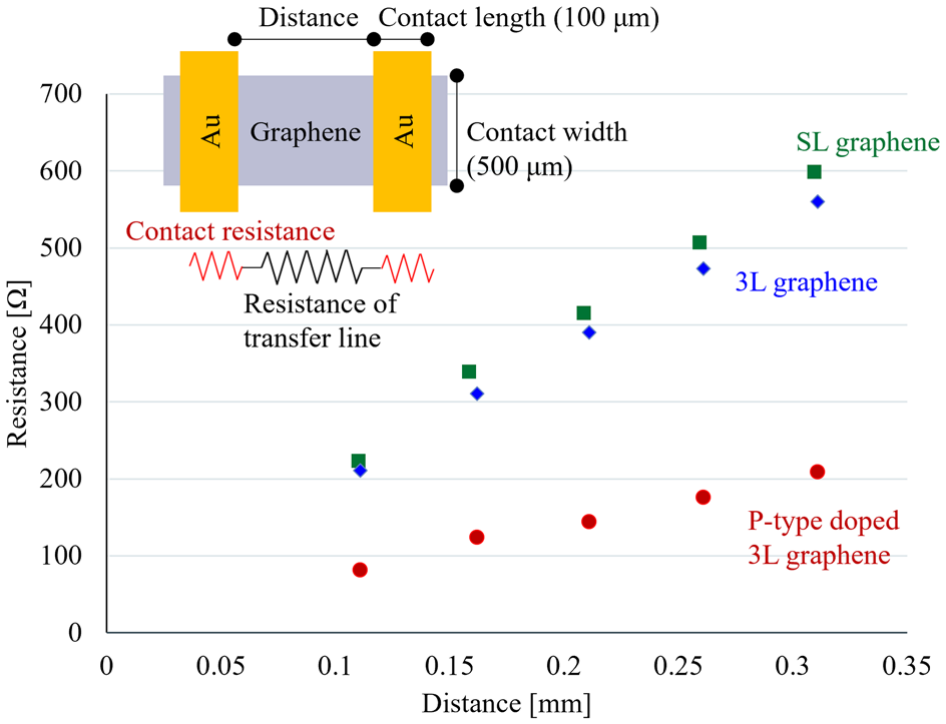
TLM for three types of graphene.

To obtain *R*_*contact*_ between graphene and Au electrode, the transfer length method (TLM) was utilized as shown in Fig. 6. In the TLM results, the Y-axis intercept refers to 2*R*_*TLM*_ between Au and the graphene^38–40^. The value of *R*_*contact*_ was determined from the contact width between graphene and Au electrode (500 μm; Fig. 6) and the width between graphene and the Au electrode in the CPW (400 μm; Fig. 3a) as shown in Table 2. Kosuga et al. reported an *R*_*contact*_ of 50 Ω for SL graphene using TLM^28^, which is not significantly different from our data (Table 2). The X-axis intercept of the straight lines in Fig. 6 represents the effective length (*L*_*t*_) that contributed to the contact between the Au electrode and graphene. *S*_*t*_ between the graphene and Au electrodes in the CPW circuit can be expressed as

$$S_{t} = L_{t} \times W$$

**Table 2:** *R*_*contact*_ of three types of graphene obtained from TLM measurements.

	SL graphene	3L graphene	P-type doped 3L graphene
*R*_*TLM*_ [Ω]	16	12	8
*R*_*contact*_ [Ω]	20	15	10
*S*_*t*_ [cm^2^]	3.4 × 10^–5^	2.8 × 10^–5^	5.3 × 10^–5^

Where *W* is the width of the CPW signal line (400 μm). P-type doped 3L graphene exhibited an increase in *St* with P-type doping (5.3 × 10^–5^ cm^2^) as shown in Table 2. This is based on the increase in the density of states (DOS) due to the lowering of the Fermi level, and as a result, P-type doped 3L graphene exhibited the lowest *R*_*contact*_ value.

### Contact capacitance ***C***_***contact***_

*C*_*contact*_ can be treated as a series circuit of the quantum capacitance of graphene (*C*_*q*_) and the geometrical capacitance (*C*_*g*_) between Au and graphene.

$$C_{contact} = \left( { \frac{1}{{C_{q} }} + \frac{1}{{C_{g} }}} \right)^{ - 1} [{\text{F}}]$$

*C*_*q*_ and *C*_*g*_ can be obtained as follows^41–43^,

$$C_{q} = \frac{{2{\text{e}}^{2} E_{F} }}{{\pi \left( {{\text{v}}_{{\text{F}}} \hbar } \right)^{2} }} \times S_{t}^{{\prime }} \,[{\text{F}}]$$

$$C_{g} = \frac{{\upvarepsilon }}{d} \times S_{t}^{{\prime }} \,[{\text{F}}]$$

where *E*_*F*_ is the Fermi energy, ${v}_{F}$ is the Fermi velocity (1 × 10^8^ cm/s), ħ is the reduced Planck's constant, and *S*_*t*_*'* is the effective contact area between graphene and Au in the AC circuit. ε is the dielectric constant, and *d* corresponds to the distance between the electrode and graphene. Here, in the DC circuit, the effective contact area between graphene and Au (*S*_*t*_) can be obtained from the TLM measurement. However, *S*_*t*_*'* in the high-frequency band is difficult to determine because the current distributions in the CPW differ from that in DC measurements due to the current crowding and its frequency dependence. Therefore, *C*_*contact*_ was treated as a fitting parameter in the analysis of S_21_, in which the measured S_21_ is compared to the simulations.

### Evaluation of S_21_

The electrical properties of the three types of graphene were reflected in an equivalent circuit model (Fig. 4) to simulate the S_21_. The simulations were performed using a 3D planar high-frequency electromagnetic software (Sonnet Lite 18.53), and after fitting *C*_*contact*_ (Fig. S3)*,* the transmission characteristics of graphene were evaluated by comparing them with the measured results. The *C*_*contact*_ fitting was performed as shown in Fig. S3. The impedance of capacitance is shown below.

$$\mathrm{Z}= \left|\frac{1}{2\pi f{C}_{contact}}\right|$$

where *f* corresponds to frequency. The *C*_*contact*_ fitting was performed at lower frequencies because *C*_*contact*_ has a greater effect on contact impedance becomes large when the frequency is close to 1 GHz. Fig. S3 shows that the S_21_ calculation results were close to the measured values and saturated when the *C*_*contact*_ is above 200 pF for SL and 3L graphene and above 1 nF for P-type doped 3L graphene. From the results, the value of *C*_*contact*_ of three types of graphene could not be uniquely determined. Therefore, for the S_21_ calculations, the *C*_*contact*_ of SL and 3L graphene was regarded as 200 pF and the *C*_*contact*_ of P-type doped 3L graphene was treated as 1 nF. Our findings suggest that the larger *C*_*contact*_ of P-type doped 3L graphene is due to the increase in quantum capacitance (*C*_*q*_) resulting from the increase in carrier density (Table 1). Hence, the value of *C*_*contact*_ may have changed as the value of *C*_*q*_ increased. This indicates that the values of *C*_*q*_ and *C*_*g*_ are comparable.

**Figure 7:**
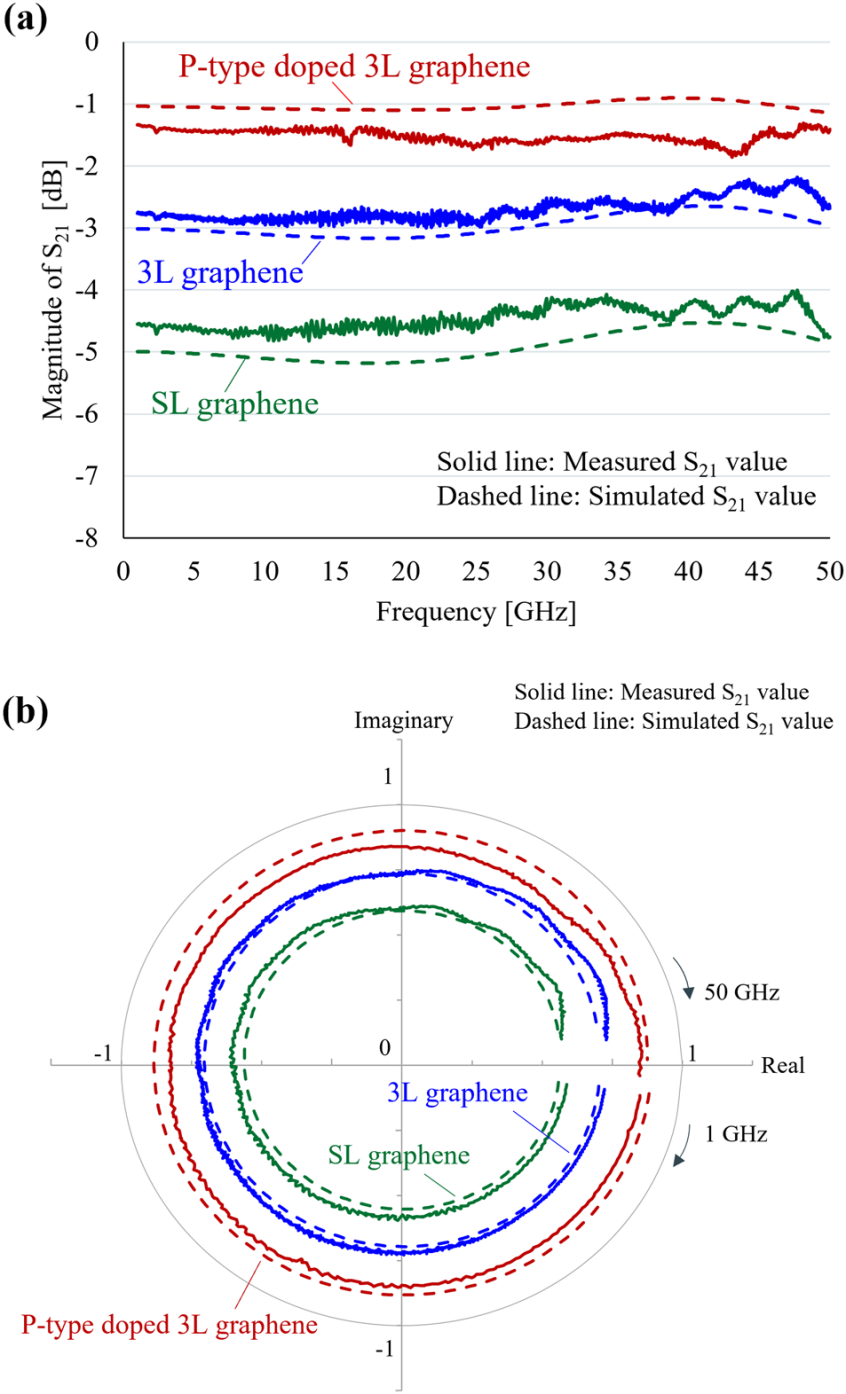
Measured (solid line) and calculated (dashed line) S_21_ for SL graphene (green), 3L graphene (blue), and P-type 3L graphene (red) from 1 to 50 GHz. (**a**) Magnitude of S_21_ for frequency. (**b**) Polar chart.

Figure 7a shows the magnitude of S_21_ versus frequency. Comparing the magnitudes of the three types of graphene, the transmission loss decreased in the order of SL, 3L, and P-type doped 3L graphene. The transmission loss was smaller with lower values of the *R*_*graphene*_ in the graphene channel (Fig. 3a), indicating that the multilayering and carrier doping of graphene are effective for reducing the transmission loss.

Figure 7b shows the experimental and calculated results of the S_21_ of the CPW in a polar chart. The polar coordinates represent the *Real* part on the X-axis and the *Imaginary* part on the Y-axis, and can be obtained from the magnitude and phase of S_21_ by the following equation. Hence, the phase and magnitude can be compared from the polar chart.

$$\mathrm{\it Real}={10}^{(\mathrm{Magnitude }\left[\mathrm{dB}\right]/ 20)}\times \mathrm{cos} \left(\frac{\mathrm{phase }\left[\mathrm{deg}.\right] \times \pi }{180 [\mathrm{deg}.]}\right)$$

$$\mathrm{\it Imaginary}={10}^{(\mathrm{Magnitude }\left[\mathrm{dB}\right]/ 20)}\times \mathrm{sin} \left(\frac{\mathrm{phase }\left[\mathrm{deg}.\right] \times \pi }{180 [\mathrm{deg}.]}\right)$$

Because the maximum value of magnitude is 0 dB, the X and Y-axes of the polar chart take values from -1 to 1. Here, magnitude and phase can be converted using the following equation.

$$\mathrm{Magnitude }\,[\mathrm{dB}]=20{\mathrm{log}}_{10}\sqrt{{Real}^{2}+{Imaginary}^{2}}$$

$$\mathrm{Phase }\,[\mathrm{deg}.]=\mathrm{Arctangent }(\frac{Imaginary }{Real})$$

The magnitude increases when the *Real* and *Imaginary* parts are large. In other words, when the value of *Real* and *Imaginary* are far from the origin in the polar chart, it means that the transmission loss is small. As shown in Fig. 7b, for both graphene samples, the calculated and experimental values were in close agreement from 1 to 50 GHz. These results indicate that both the magnitude and the phase were accurate, thus validating the design policy of this equivalent circuit model. Furthermore, the results indicate that the impedance of graphene channel in the CPW (Fig. 3a) can be expressed only in terms of resistance from 1 to 50 GHz frequency, as discussed in the previous section. It is also demonstrated that the introduction of open stubs brings the measured values closer to the calculated values in Fig. S4, and the open stubs was necessary for the equivalent circuit model.

**Figure 8:**
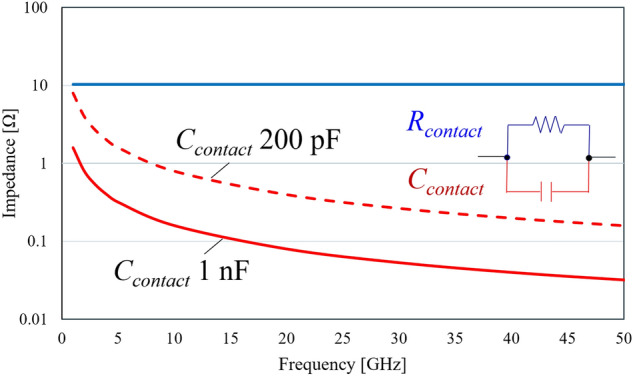
Calculated frequency dependence of impedance between graphene and Au electrode. *R*_*contact*_ (10 Ω) of P-type doped 3L graphene (blue line), *C*_*contact*_ (1 nF) of P-type doped 3L graphene (solid red line) and *C*_*contact*_ (200 pF) of 3L graphene (dashed red line) from 1 to 50 GHz.

The Au and graphene contacts of the CPW used in this study ware represented by a parallel circuit of *R*_*contact*_ and *C*_*contact*_. Figure 8 shows the calculation and comparison of the impedance of *R*_*contact*_ and *C*_*contact*_ versus frequency. In the frequency range from 1 to 50 GHz, the impedance of *C*_*contact*_ is clearly smaller, indicating that the contact impedance is dominated by capacitance^10^. Awan et al.’s study^29^, which was used as a reference for constructing the equivalent circuit model, evaluated the transmission characteristic of CPW with SL graphene but did not assume that capacitance is the dominant factor in contact impedance. The value of *C*_*contact*_ in Awan et al.’s experiment was set at 0.12 pF, which is smaller than our value (*C*_*contact*_ > 200 pF). This is because the contact area between the Au electrode and graphene in our study (Fig. 3a) is larger than in Awan et al*.* (100 μm^2^). Thus, these results indicated that the large contact area between the Au and graphene enabled the *C*_*contact*_ to be the dominant factor in the contact impedance.

Our research clarified the transmission characteristics of graphene for the 5G frequency by experimentally measuring the S_21_ of the CPW using the graphene at 1 –50 GHz and comparing them with the constructed equivalent circuit model of the CPW. To our knowledge, our study is the first compare the S_21_ of various graphene materials with different electrical properties while maintaining transparency. From this comparison, we found that the graphene channel in the CPW can be expressed only in terms of resistance. In addition, multilayering and carrier doping are effective in reducing transmission loss of the graphene, and the contact impedance can be reduced by increasing the contact area between Au and graphene. These are important findings for the design of transparent 5G antenna devices using graphene.

It is evident that graphene will continue to be studied to lower resistivity, increase mobility, and enable processes for larger area, toward a variety of device applications. Our study is one step in that direction, and it has provided valuable insights not only for designing transparent 5G antennas with graphene but also for the design of other devices such as microwave ring resonators, absorber, photodetectors, and terahertz devices using the graphene.

## Conclusion

We have fabricated CPWs using three types of graphene and measured the S_21_ from 1 to 50 GHz. We also measured the optical and electrical characteristics of the three types of graphene, built equivalent circuit models, and calculated. The agreement between the simulated S_21_ and measured S_21_ supports the validity of the constructed equivalent circuit model, in addition, the contact impedance value and the transmission loss of graphene channel were clarified. The comparison of the S_21_ of the three types of graphene shows that carrier doping and multilayering of graphene are effective in reducing transmission loss while maintaining the transparency of graphene in the operated 5G frequency. The results provide design guidelines for the introduction of graphene as a transparent conductive material in 5G antennas to replace existing materials such as meshed Cu, metal nanowires, and ITO films. Increasing the contact area between Au and graphene was also found to be effective in reducing contact impedance. In designing 5G antennas, it is important to connect the device to the power feed with minimal losses, and our experimental results show that a large contact area between the graphene and electrode is an important consideration as it reduces contact losses.

Our findings provide insights into the design of millimeter-wave devices using graphene and, furthermore, provide the basis for the application of graphene as a radiating element of transparent 5G antennas.

## Methods

**Figure 9:**
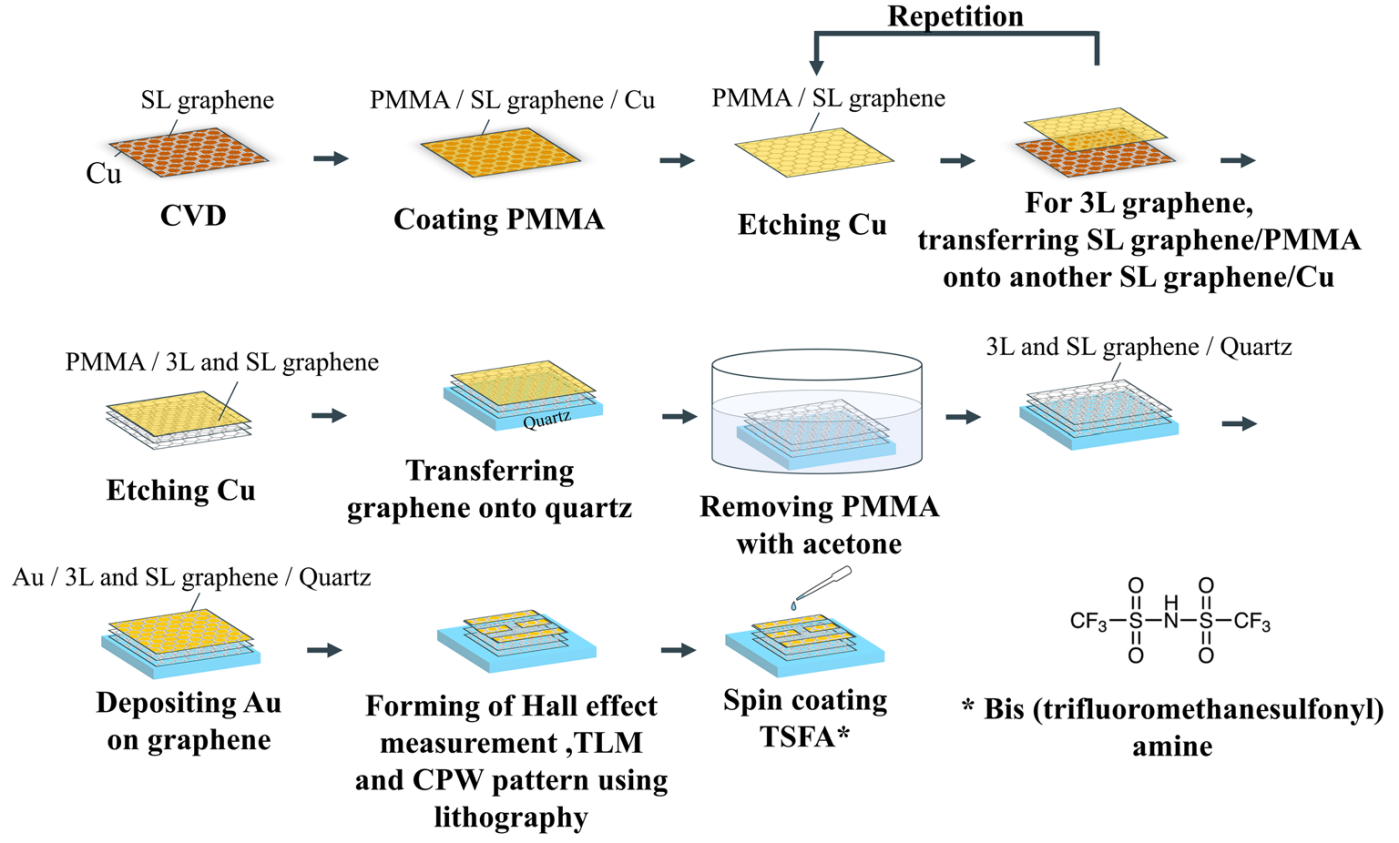
Graphene and device fabricating processes.

Figure 9 shows a series of processes from graphene growth to device fabrication.

### CVD growth and graphene stacking

To grow graphene as a uniform monolayer, Cu foil was selected as a catalyst substrate. Before graphene growth, the Cu foil was annealed at 1000 °C for 30 min in H_2_ (20 sccm) atmosphere for cleaning. Graphene was grown by low-pressure CVD on the Cu foil using H_2_ and CH_4_ gas. The CVD growth proceeded for 30 min under H_2_ (20 sccm) and CH_4_ (2 sccm) flows at 1000 °C. For transfer processes, PMMA (Aldrich, M.W. = 996,000) in an ethyl lactate solution (4 wt%) was spin-coated onto graphene as a supporting layer and cured at 180 °C for 1 min.

The PMMA was wiped off with acetone during spin-coating because it also spread around to the back side of Cu foil. In the CVD process, graphene grew on both sides of the Cu foil, causing subsequent Cu etching failure. Therefore, oxygen plasma treatment (O_2_:30 sccm, power: 20 W, time: 60 s) was performed on the opposite side of the Cu foil from the side where the PMMA film was formed to remove the unwanted graphene. Then the PMMA coated SL graphene sheet was obtained by etching the Cu foil. To form SL graphene on a substrate, the PMMA-coated SL graphene sheet (size: 10 × 10 mm^2^) was transferred onto a quartz substrate (size: 20 × 20 mm^2^, thickness: 1 mm), and the PMMA was removed by immersing it in an acetone for 12 h. The 3L graphene samples were obtained by a layer-by-layer process in which a PMMA-coated SL graphene sheet was repeatedly transferred to another graphene grown on a Cu foil sample. The rest of the process was the same as that for fabricating SL graphene: etching Cu, transferring to the quartz substrate, and removing PMMA to obtain the 3L graphene on the quartz sample.

### Device fabrication

For the device fabrication of TLM, Hall effect measurement, and the CPW, Au was deposited onto three types of graphene samples as electrode materials. The Au electrodes for the Hall effect measurement sample were deposited in vacuum using resistance heating at a rate of 1 Åm/s, and the resulting film thickness was 100 nm. In addition, for the TLM and CPW samples, Au electrodes were prepared by electron beam evaporation in vacuum at a rate of 10 Åm/s and deposited to a thickness of 500 nm.

Electrode patterning for each device formation was performed in two steps. The first process is to remove unwanted Au and graphene, and the second step is to remove Au on the graphene channel area. For the first process, a photo resist (Merck, AZ5214-E) was spin-coated onto the Au and cured at 90 °C for 1.5 min. After UV exposure, the samples were immersed in a developing solution (Merck, AZ 300MF DEVELOPER (2.38%)) to remove the resist from areas other than the electrode patterns. The samples were then immersed in Au etchant (Kanto Chemical, AURUM303) for 40 s to form the electrode pattern. Finally, the resist on the electrode pattern was removed using a remover (Merck, AZ remover 700), followed by oxygen plasma treatment (O_2_:30 sccm, power:100 W, time : 120 s) to completely remove the resist and unwanted graphene residue . The second process is the formation of the graphene channel. Resist coating, UV exposure using a mask for channel formation, immersion in the developing solution, dipping in the Au etchant, followed by immersion in the developing solution again, and oxygen plasma treatment were performed as in the first process. Through these processes, various devices could be fabricated with graphene.

The P-type doped 3L graphene was obtained by dropping TFSA (TCI, B2541) in 1-butanol solution (50 mM) onto 3L graphene, allowing it to soak for 1 min at room temperature in air atmosphere, and then spin coating it^10^.

### Supplementary Information

Supplementary Figures.

## Data Availability

The datasets analyzed in the current study are available from the corresponding authors upon request.
