# Supplementary material for: Evaluation of transmission characteristics of CVD-grown graphene and effect of tuning electrical properties of graphene up to 50 GHz
Source: Sci Rep. 2023 Aug 24;13:13878. doi: 10.1038/s41598-023-40942-8 (PMC10449821; doi:10.1038/s41598-023-40942-8)
Supplement: Supplementary file 1 — Supplementary Figures. [file 41598_2023_40942_MOESM1_ESM.docx]

Supplementary information

Evaluation of impedance characteristics of CVD-grown graphene and effect of tuning of electrical properties of graphene up to 50 GHz

Ryota Okuda *et al.*

Corresponding author: Ryota Okuda ([Ryota.okuda@agc.com](mailto:Ryota.okuda@agc.com)

Supplementary figures


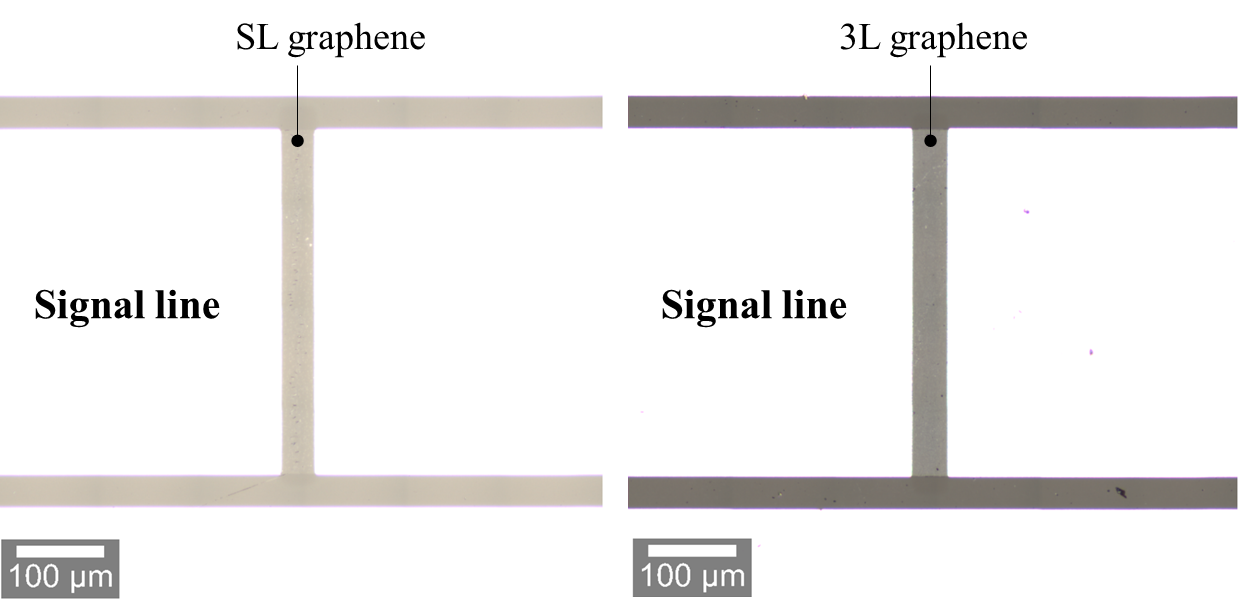
Fig. S1 shows the optical microscope images of the graphene channel with SL and 3L graphene in the CPW used for the measurement. Significant damage was not observed in Fig. S1.

**Figure S1.** Optical microscope images of CPW with SL and 3Lgraphene. P-type doped 3L graphene was omitted because it corresponds to the same sample as the 3L graphene.

Fig. S2 shows a photograph of a CPW sample after measurements of the S_21_ characteristics. Since the conductor was shaved at the position where the probe came in contact, the distance between the contact position and the edge of the transmission line was reflected in the equivalent circuit model as the length of the stub (Fig. 4).


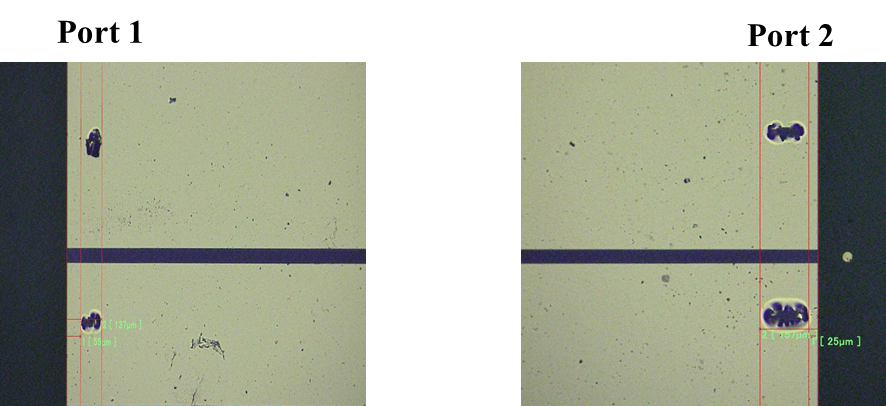


**Figure S2.** Microscopic measurement of the distance from the touchdown position to the end of the transmission line. The photo shows a sample of measurement using p-type doped 3L graphene. The lengths of the stubs were 137 μm and 157 μm, respectively.

Fitting of *C_contact_* was performed on a polar chart that reflects the phase and amplitude components (Fig. S2). The impedance (Z) of capacitance is shown below.

$$Z= \left| \frac{1}{2\pi fC_{contact}} \right|$$

where *f* corresponds to frequency. The *C_contact_* fitting was performed at lower frequencies because *C_contact_* has a greater effect on contact impedance becomes large when the frequency is close to 1 GHz. Fig. S3 shows that the S_21_ calculation results were close to the measured values and saturated when the *C_contact_* is above 200 pF for SL and 3L graphene and above 1 nF for P-type doped 3L graphene. From the results, the value of *C_contact_* of three types of graphene could not be uniquely determined. Therefore, for the S_21_ calculations, the *C_contact_* of SL and 3L graphene was regarded as 200 pF and the *C_contact_* of P-type doped 3L graphene was treated as 1 nF.


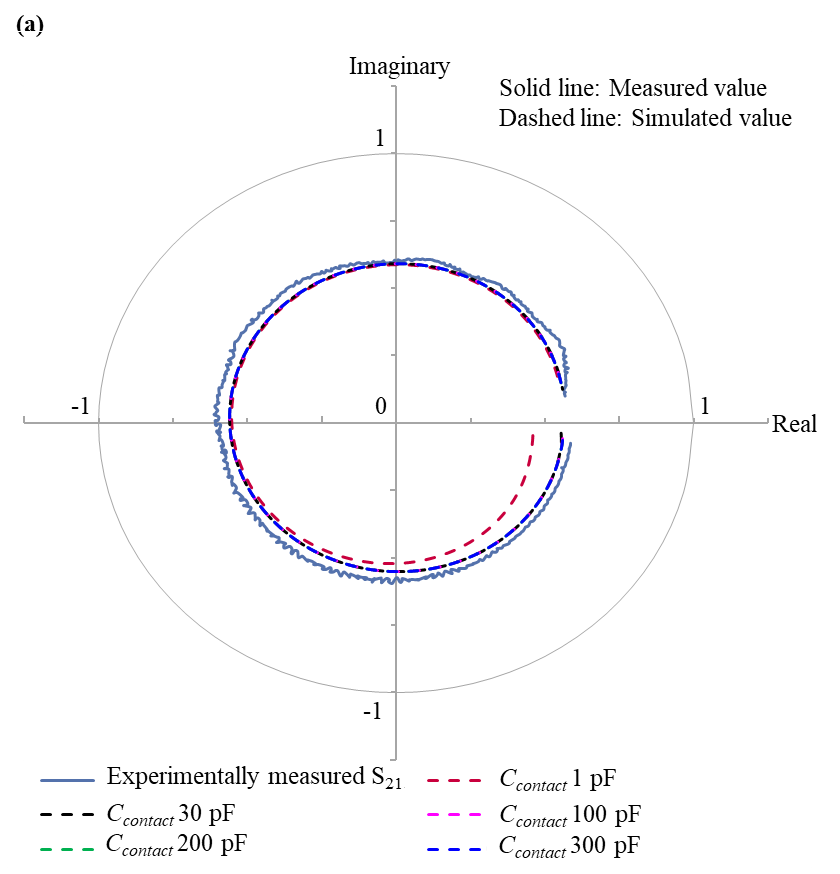


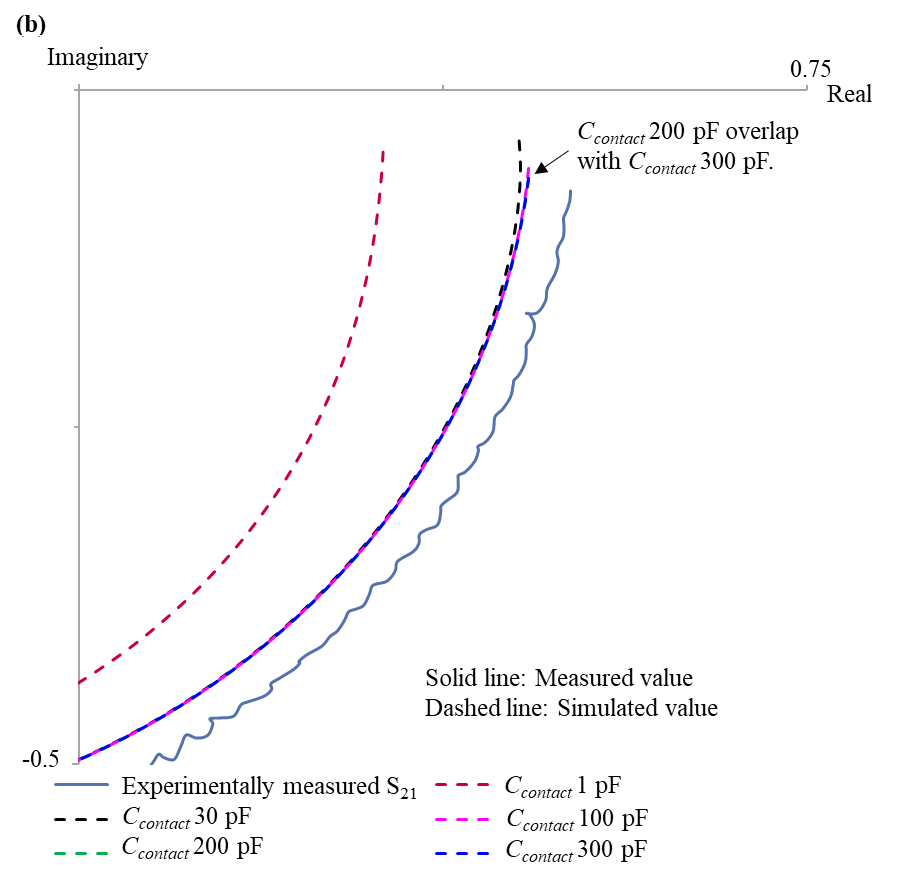


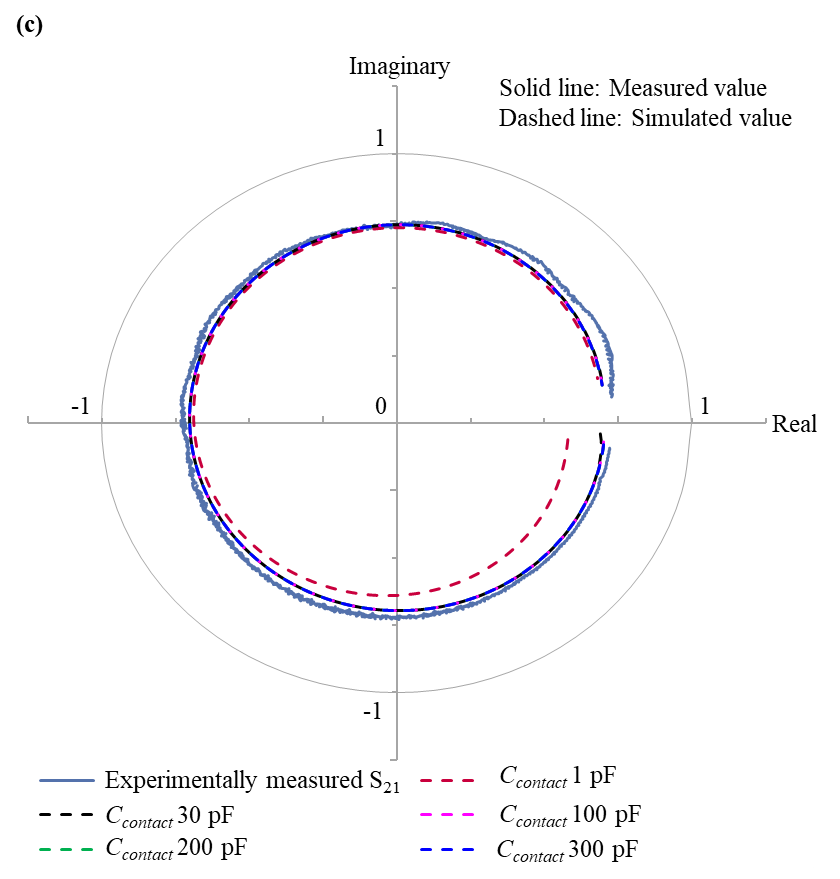


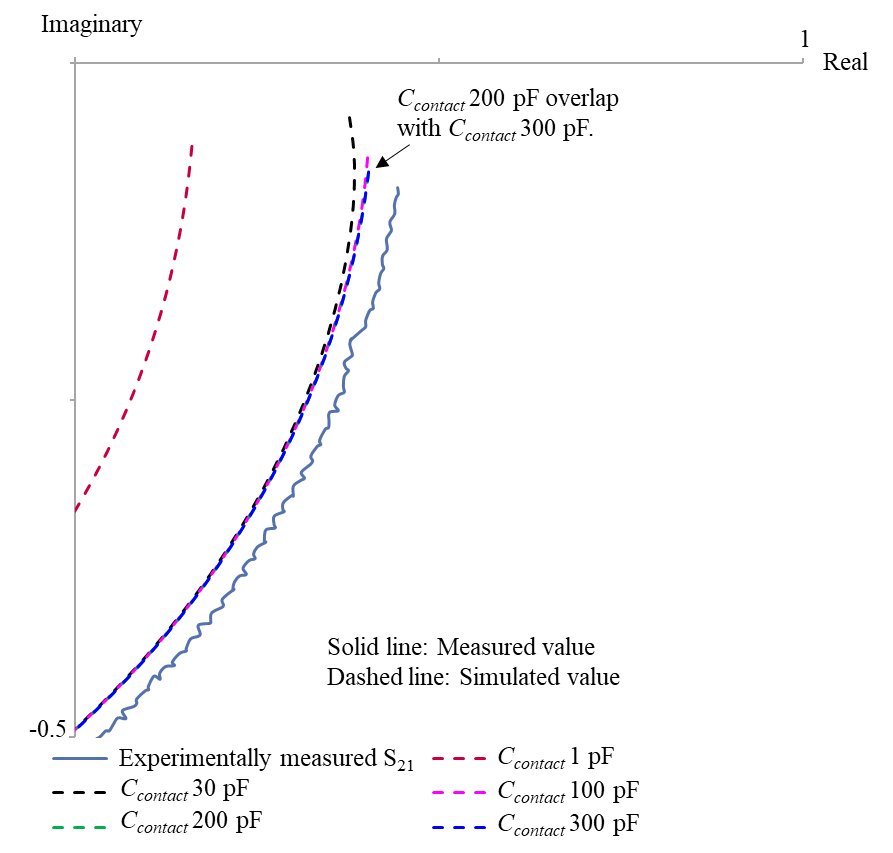


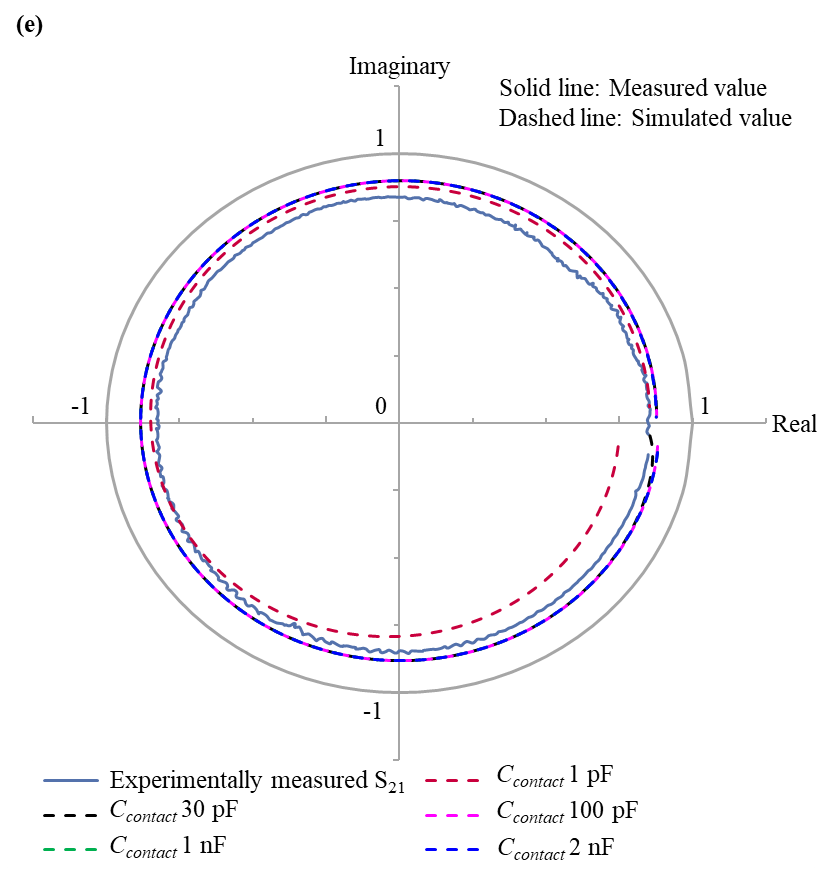

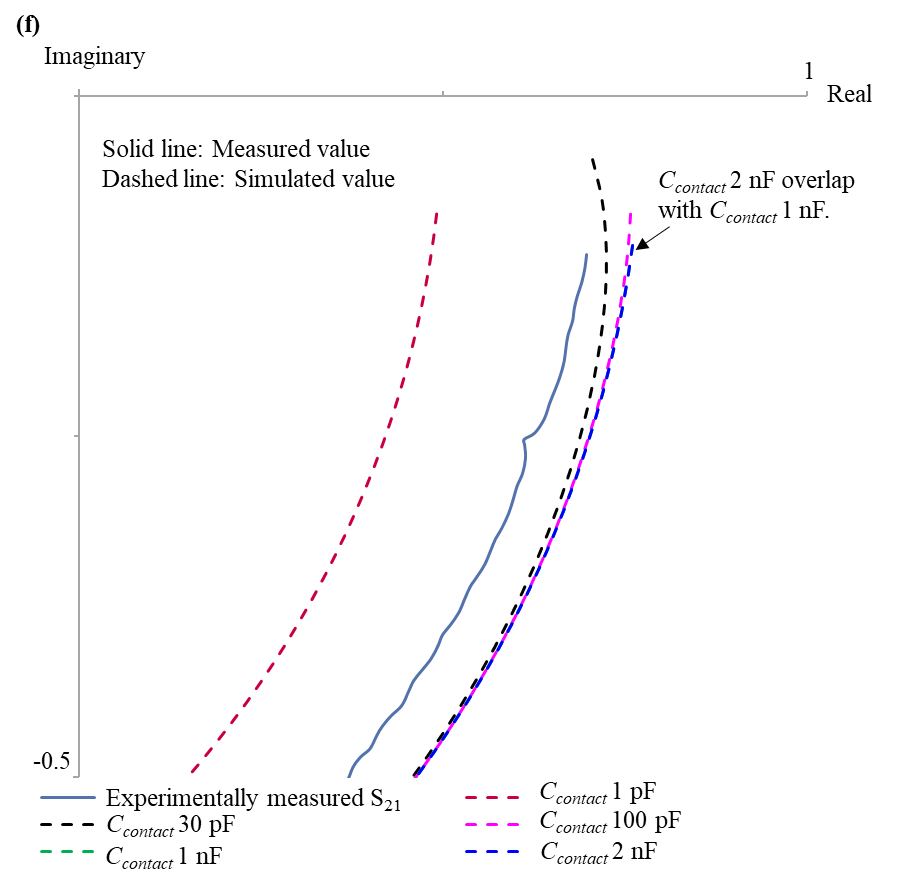


**Figure S3.** Fitting results of *C_contact_* using Sonnet Lite 18.53　for 3 types of graphene and Au contacts. a) is the fitting result for SL graphene, and b) is the result extended in the low frequency range. c) is the fitting result for 3L graphene, and d) is the result extended in the low frequency range. e) is the fitting result for P-type doped 3L graphene, and f) is the result extended in the low frequency range.

The effect of the stubs included in the equivalent circuit of Fig. 4 was also compared on a polar chart for the S_21_ characteristic (Fig. S3). The stub length was set to 137 μm and 157 μm based on actual measurements, and input into the equivalent circuit model as a transmission line with a characteristic impedance of 50 Ω.

Fig S3 shows that the calculated result of S_21_ without stubs (red dash line) has a larger phase rotation than the measured value (blue solid line) and the calculated result of S_21_ including stubs (green dash line). This is because the probe should ideally be placed at the end of the electrode, and the calculated line length is longer than the measured length. On the other hand, the calculated result for S_21_ including the stubs were closer to the measured values. Therefore, it is effective to design the equivalent circuit model (Fig. 4) with stubs.


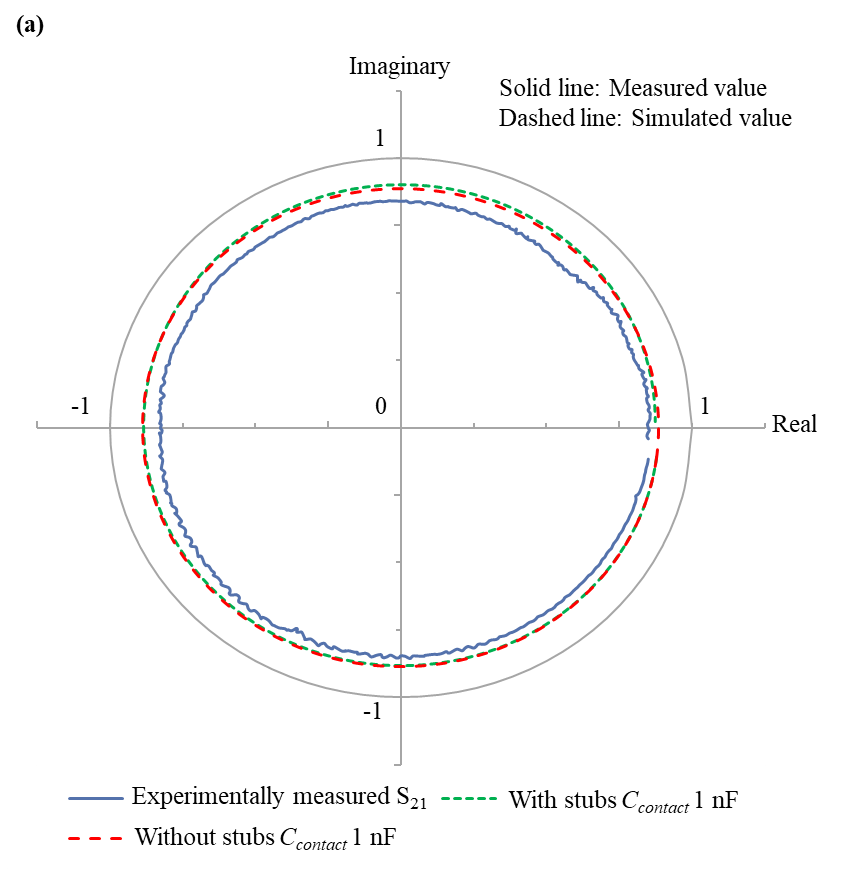


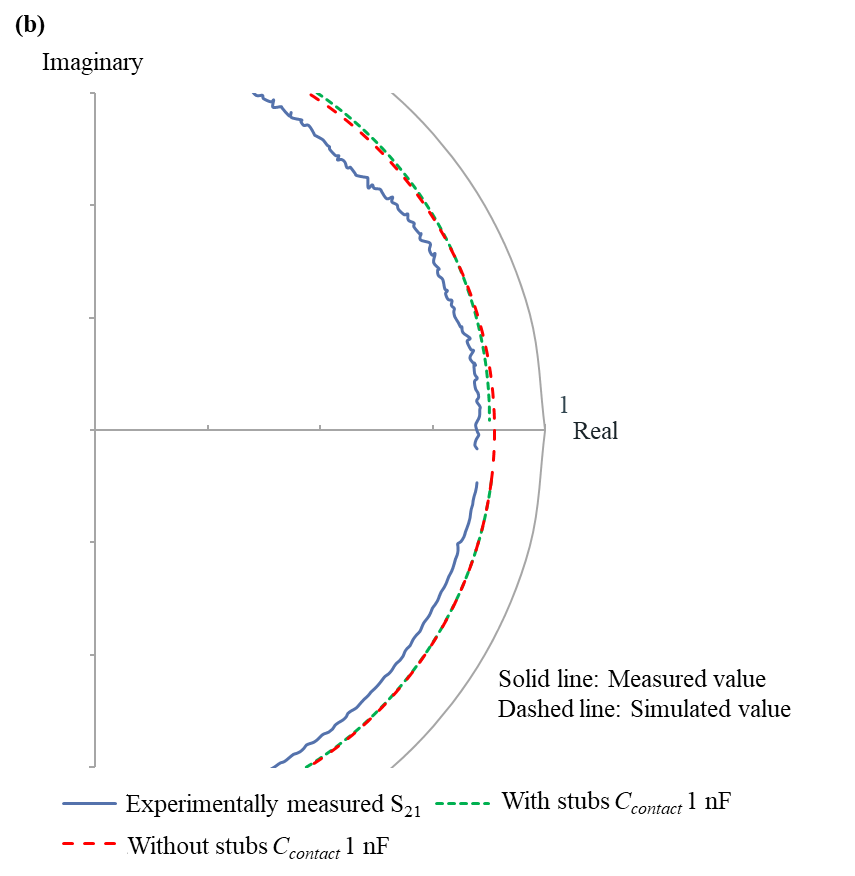


**Figure S4.** Measured and simulated results of S_21_ characteristics in the polar chart. a) is the fitting result for P-type doped 3L graphene, and b) is the extended result. Comparison was made with measured values (blue solid line), S_21_ calculated values with stubs (green dash line), and S_21_ calculated values without stubs (red dash line).
